# NdO(NO_3_)

**DOI:** 10.1107/S1600536808036295

**Published:** 2008-11-13

**Authors:** Ya-Feng Li, Li Jin, Dan-Ping Li, Long Zhang

**Affiliations:** a School of Chemical Engineering, Changchun University of Technology, Changchun 130012, People’s Republic of China

## Abstract

The title compound, neodymium(III) oxide nitrate, which is isostructural with LaO(NO_3_), arose from a solvothermal reaction. The Nd ion (site symmetry *m*) is ten-coordinated by eight O atoms of NO_3_ groups and two μ_2_-oxide ions. A three-dimensional structure is constructed by the inter­connection of NdO_10_ polyhedra. The oxide ion and the N atom and one of the nitrate O atoms possess site symmetry *m*.

## Related literature

For background, see: Gobichon *et al.* (1997 ▶); Guillou *et al.* (1994 ▶). For an isostructural compound, see: Zhang *et al.* (2004 ▶).

## Experimental

### 

#### Crystal data

NdO(NO_3_)
*M* *_r_* = 222.25
Orthorhombic, 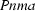

*a* = 7.5233 (15) Å
*b* = 5.1618 (10) Å
*c* = 8.7157 (17) Å
*V* = 338.46 (11) Å^3^
*Z* = 4
Mo *K*α radiation
μ = 15.19 mm^−1^
*T* = 293 (2) K
0.16 × 0.14 × 0.12 mm

#### Data collection

Rigaku R-AXIS RAPID diffractometer
Absorption correction: multi-scan (*ABSCOR*; Higashi, 1995 ▶) *T*_min_ = 0.107, *T*_max_ = 0.158
2962 measured reflections
410 independent reflections
405 reflections with *I* > 2σ(*I*)
*R*_int_ = 0.034

#### Refinement

*R*[*F* ^2^ > 2σ(*F* ^2^)] = 0.024
*wR*(*F* ^2^) = 0.068
*S* = 1.81
410 reflections
34 parameters
30 restraints
Δρ_max_ = 1.02 e Å^−3^
Δρ_min_ = −1.42 e Å^−3^

### 

Data collection: *PROCESS-AUTO* (Rigaku, 2002 ▶); cell refinement: *PROCESS-AUTO*; data reduction: *CrystalStructure* (Rigaku, 2002 ▶); program(s) used to solve structure: *SHELXS97* (Sheldrick, 2008 ▶); program(s) used to refine structure: *SHELXL97* (Sheldrick, 2008 ▶); molecular graphics: *DIAMOND* (Brandenburg, 2000 ▶); software used to prepare material for publication: *SHELXL97*.

## Supplementary Material

Crystal structure: contains datablocks I, global. DOI: 10.1107/S1600536808036295/hb2833sup1.cif

Structure factors: contains datablocks I. DOI: 10.1107/S1600536808036295/hb2833Isup2.hkl

Additional supplementary materials:  crystallographic information; 3D view; checkCIF report

## Figures and Tables

**Table 1:** Selected bond lengths (Å)

Nd1—O2^i^	2.434 (5)
Nd1—O2	2.458 (5)
Nd1—O3^ii^	2.6362 (12)
Nd1—O1	2.694 (3)
Nd1—O1^iii^	2.719 (4)
Nd1—O1^ii^	2.826 (4)

Symmetry codes: (i) 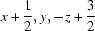; (ii) 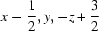; (iii) 
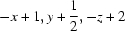
.

**Fig. 1.**
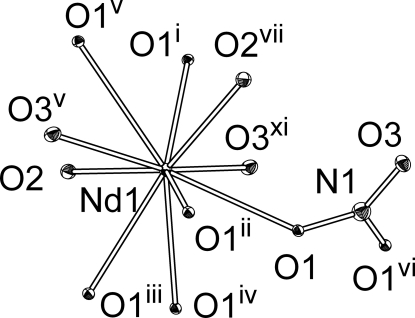
A fragment of the structure of (I), showing the Nd coordination sphere and displacement ellipsoids at the 50% probability level. [Symmetry codes: (i) -1/2 + x, y, 1.5 - z; (ii) x, 0.5 - y, z; (iii) 1 - x, 1/2 + y, 2 - z; (iv) 1 - x, -y, 2 - z; (v) -1/2 + x, 0.5 - y, 1.5 - z; (vi) x, -0.5 - y, z; (vii) 1/2 + x, 0.5 - y, 1.5 - z.]

**Fig. 2.**
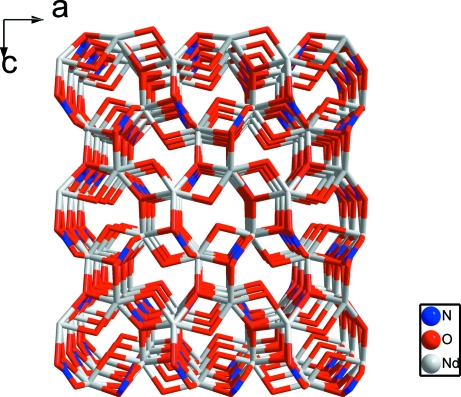
A packing diagram for (I), viewed along [010].

## supplementary crystallographic information

### Comment

The lanthanide nitrates are not only applied for separation of the lanthanide elements but also widely utilized as the precursor of organic or inorganic synthesis.  Thus, a large number of lanthanide nitrates are structurally determinated besides a few anhydrous examples (Guillou, *et al.*, 1994; Zhang, *et al.*, 2004; Gobichon, *et al.*, 1997). In this work, the title compound, (I), an anhydrous neodymium oxide nitrate, was unexpectedly obtained under solvothermal conditions in a mixed solvent of H_2_O and DMF.

The asymmetric unit of (I) is consisted of 0.5 N d, 0.5 O and 0.5 NO_3_ (Fig. 1). All oxygen atoms of NO_3_ group are coordinated to the Nd ions. Two oxygen atoms of nitrate group (O1 and O1^vi^) are coordinated to three different Nd ions with Nd—O distances in the range of 2.694–2.826 A°, and the last one (O3) is coordinated to two different Nd ions with Nd—O distance of 2.636 A° (Table 1). A µ_2_-O (O2) exists in the structure of (I) with Nd—O distances of 2.434 and 2.458 A° and corresponding Nd—O2—Nd bond angles of 110.72°. These two Nd—O distances are significantly shorter than the others Nd—O distances.  Then, the linkages of two adjacent Nd ions are in two modes, of which one is *via* Nd-µ_2_-O—Nd bonds with Nd—Nd distance of 4.0254 (8)A° and the other *via* Nd—O(NO_3_)-Nd bonds. A three-dimensional framework constructed by the interconnections of NdO_10_ polyhedra is shown in Fig. 2.

There are two different structures with the same molecular formula of LnONO_3_, such as LnONO_3_(Ln=Y, La) in the P_4_/*mmm* space group and LaONO_3_ in *Pnma* space group. In this work, NdONO_3_ is the isostructural compound of the reported LaONO_3_ (Zhang, *et al.*, 2004).

### Experimental

Isonicotine (0.123 g, 1.0 mmol) was added to a mixed solution of 5 ml H_2_O/3 ml DMF. After being stirred for 5 h, the isonicotine was partially dissovled with pH = 6.0. Then, Nd(NO_3_)_3_^.^6H_2_O (0.220 g, 0.5 mmol) was added and stirred for 7 h. The molar ratio of Nd(NO_3_)_3_^.^6H_2_O: isonicotine was 1:2. Finally, the solution with pH = 7.0 was sealed into 23 ml autoclave and heated up to 438 K for 4 days. After naturally cooling to room temperature, colourless prisms of (I) were obtained.

### Crystal data

**Table:**

NdO(NO_3_)	*F*_000_ = 396
*M**_r_* = 222.25	*D*_x_ = 4.362 Mg m^−^^3^
Orthorhombic, *P**n**m**a*	Mo *K*α radiation λ = 0.71073 Å
Hall symbol: -P 2ac 2n	Cell parameters from 2000 reflections
*a* = 7.5233 (15) Å	θ = 3.6–27.0º
*b* = 5.1618 (10) Å	µ = 15.19 mm^−^^1^
*c* = 8.7157 (17) Å	*T* = 293 (2) K
*V* = 338.46 (11) Å^3^	Prism, colourless
*Z* = 4	0.16 × 0.14 × 0.12 mm

### Data collection

**Table:**

Rigaku R-AXIS RAPID diffractometer	410 independent reflections
Radiation source: fine-focus sealed tube	405 reflections with *I* > 2σ(*I*)
Monochromator: graphite	*R*_int_ = 0.034
Detector resolution: 10.00 pixels mm^-1^	θ_max_ = 27.0º
*T* = 293(2) K	θ_min_ = 3.6º
ω scans	*h* = −9→8
Absorption correction: multi-scan(ABSCOR; Higashi, 1995)	*k* = −5→6
*T*_min_ = 0.107, *T*_max_ = 0.158	*l* = −11→11
2962 measured reflections	

### Refinement

**Table:**

Refinement on *F*^2^	Primary atom site location: structure-invariant direct methods
Least-squares matrix: full	Secondary atom site location: difference Fourier map
*R*[*F*^2^ > 2σ(*F*^2^)] = 0.024	*w* = 1/[σ^2^(*F*_o_^2^) + (0.0285*P*)^2^] where *P* = (*F*_o_^2^ + 2*F*_c_^2^)/3
*wR*(*F*^2^) = 0.068	(Δ/σ)_max_ = 0.002
*S* = 1.81	Δρ_max_ = 1.02 e Å^−^^3^
410 reflections	Δρ_min_ = −1.42 e Å^−^^3^
34 parameters	Extinction correction: none
30 restraints	

### Special details

**Table:**

Geometry. All e.s.d.'s (except the e.s.d. in the dihedral angle between two l.s. planes) are estimated using the full covariance matrix. The cell e.s.d.'s are taken into account individually in the estimation of e.s.d.'s in distances, angles and torsion angles; correlations between e.s.d.'s in cell parameters are only used when they are defined by crystal symmetry. An approximate (isotropic) treatment of cell e.s.d.'s is used for estimating e.s.d.'s involving l.s. planes.
Refinement. Refinement of *F*^2^ against ALL reflections. The weighted *R*-factor *wR* and goodness of fit *S* are based on *F*^2^, conventional *R*-factors *R* are based on *F*, with *F* set to zero for negative *F*^2^. The threshold expression of *F*^2^ > σ(*F*^2^) is used only for calculating *R*-factors(gt) *etc*. and is not relevant to the choice of reflections for refinement. *R*-factors based on *F*^2^ are statistically about twice as large as those based on *F*, and *R*- factors based on ALL data will be even larger.

### Fractional atomic coordinates and isotropic or equivalent isotropic displacement parameters (Å^2^)

**Table:**

	*x*	*y*	*z*	*U*_iso_*/*U*_eq_	
Nd1	0.35352 (4)	0.2500	0.83222 (4)	0.0055 (2)	
O1	0.6501 (4)	−0.0288 (7)	0.8846 (4)	0.0047 (7)	
O2	0.0359 (6)	0.2500	0.8985 (6)	0.0080 (10)	
O3	0.7902 (7)	−0.2500	0.6964 (6)	0.0088 (10)	
N1	0.6934 (10)	−0.2500	0.8195 (7)	0.0120 (13)	

### Atomic displacement parameters (Å^2^)

**Table:**

	*U*^11^	*U*^22^	*U*^33^	*U*^12^	*U*^13^	*U*^23^
Nd1	0.0051 (3)	0.0052 (3)	0.0061 (3)	0.000	0.00018 (11)	0.000
O1	0.0054 (10)	0.0043 (10)	0.0045 (10)	0.0002 (7)	0.0004 (7)	−0.0001 (8)
O2	0.0072 (12)	0.0091 (12)	0.0076 (13)	0.000	−0.0001 (9)	0.000
O3	0.0091 (13)	0.0088 (13)	0.0086 (12)	0.000	0.0022 (9)	0.000
N1	0.0121 (15)	0.0120 (15)	0.0121 (15)	0.000	−0.0005 (9)	0.000

### Geometric parameters (Å, °)

**Table:**

Nd1—O2^i^	2.434 (5)	Nd1—Nd1^ii^	4.0254 (8)
Nd1—O2	2.458 (5)	Nd1—Nd1^i^	4.0254 (8)
Nd1—O3^ii^	2.6362 (12)	O1—N1	1.316 (5)
Nd1—O3^iii^	2.6362 (12)	O1—Nd1^vi^	2.719 (4)
Nd1—O1^iv^	2.694 (3)	O1—Nd1^i^	2.826 (4)
Nd1—O1	2.694 (3)	O2—Nd1^ii^	2.434 (5)
Nd1—O1^v^	2.719 (4)	O3—N1	1.297 (8)
Nd1—O1^vi^	2.719 (4)	O3—Nd1^i^	2.6362 (12)
Nd1—O1^ii^	2.826 (4)	O3—Nd1^viii^	2.6362 (12)
Nd1—O1^vii^	2.826 (4)	N1—O1^ix^	1.316 (5)
			
O2^i^—Nd1—O2	137.90 (13)	O3^ii^—Nd1—O1^ii^	48.73 (13)
O2^i^—Nd1—O3^ii^	91.36 (11)	O3^iii^—Nd1—O1^ii^	109.73 (13)
O2—Nd1—O3^ii^	81.18 (12)	O1^iv^—Nd1—O1^ii^	146.51 (6)
O2^i^—Nd1—O3^iii^	91.36 (11)	O1—Nd1—O1^ii^	106.85 (12)
O2—Nd1—O3^iii^	81.18 (12)	O1^v^—Nd1—O1^ii^	144.63 (7)
O3^ii^—Nd1—O3^iii^	156.5 (2)	O1^vi^—Nd1—O1^ii^	112.81 (4)
O2^i^—Nd1—O1^iv^	70.92 (12)	O2^i^—Nd1—O1^vii^	75.69 (11)
O2—Nd1—O1^iv^	139.92 (10)	O2—Nd1—O1^vii^	68.33 (11)
O3^ii^—Nd1—O1^iv^	133.51 (14)	O3^ii^—Nd1—O1^vii^	109.73 (13)
O3^iii^—Nd1—O1^iv^	69.06 (14)	O3^iii^—Nd1—O1^vii^	48.73 (13)
O2^i^—Nd1—O1	70.92 (12)	O1^iv^—Nd1—O1^vii^	106.85 (12)
O2—Nd1—O1	139.92 (10)	O1—Nd1—O1^vii^	146.51 (6)
O3^ii^—Nd1—O1	69.06 (14)	O1^v^—Nd1—O1^vii^	112.81 (4)
O3^iii^—Nd1—O1	133.51 (14)	O1^vi^—Nd1—O1^vii^	144.63 (7)
O1^iv^—Nd1—O1	64.57 (15)	O1^ii^—Nd1—O1^vii^	61.23 (15)
O2^i^—Nd1—O1^v^	139.04 (10)	Nd1^ii^—Nd1—Nd1^i^	138.29 (2)
O2—Nd1—O1^v^	77.12 (12)	N1—O1—Nd1	126.5 (4)
O3^ii^—Nd1—O1^v^	119.67 (13)	N1—O1—Nd1^vi^	91.7 (3)
O3^iii^—Nd1—O1^v^	70.90 (13)	Nd1—O1—Nd1^vi^	111.71 (11)
O1^iv^—Nd1—O1^v^	68.29 (11)	N1—O1—Nd1^i^	91.1 (3)
O1—Nd1—O1^v^	94.51 (7)	Nd1—O1—Nd1^i^	93.62 (12)
O2^i^—Nd1—O1^vi^	139.04 (10)	Nd1^vi^—O1—Nd1^i^	145.72 (12)
O2—Nd1—O1^vi^	77.12 (12)	Nd1^ii^—O2—Nd1	110.73 (19)
O3^ii^—Nd1—O1^vi^	70.90 (13)	N1—O3—Nd1^i^	100.35 (11)
O3^iii^—Nd1—O1^vi^	119.67 (13)	N1—O3—Nd1^viii^	100.35 (11)
O1^iv^—Nd1—O1^vi^	94.51 (7)	Nd1^i^—O3—Nd1^viii^	156.5 (2)
O1—Nd1—O1^vi^	68.29 (11)	O3—N1—O1	119.7 (3)
O1^v^—Nd1—O1^vi^	49.66 (16)	O3—N1—O1^ix^	119.7 (3)
O2^i^—Nd1—O1^ii^	75.69 (11)	O1—N1—O1^ix^	120.4 (6)
O2—Nd1—O1^ii^	68.33 (12)		

Symmetry codes: (i) *x*+1/2, *y*, −*z*+3/2; (ii) *x*−1/2, *y*, −*z*+3/2; (iii) *x*−1/2, *y*+1, −*z*+3/2; (iv) *x*, −*y*+1/2, *z*; (v) −*x*+1, *y*+1/2, −*z*+2; (vi) −*x*+1, −*y*, −*z*+2; (vii) *x*−1/2, −*y*+1/2, −*z*+3/2; (viii) *x*+1/2, *y*−1, −*z*+3/2; (ix) *x*, −*y*−1/2, *z*.
